# Establishment of recurrent laryngeal nerve injury animal models using intraoperative neural monitoring: SD rat models

**DOI:** 10.3389/fendo.2026.1856420

**Published:** 2026-07-29

**Authors:** Yuqing Gao, Huixin Li, Tie Wang, Yishen Zhao, Shijie Li

**Affiliations:** Department of Thyroid Surgery, China-Japan Union Hospital of Jilin University, Changchun, China

**Keywords:** animal model, intraoperative neural monitoring, Recurrent laryngeal nerve injury, Sprague-Dawley (SD) rats, thyroid

## Abstract

**Objective:**

To reproduce the Sprague Dawley (SD) rat model of recurrent laryngeal nerve (RLN) injury using intraoperative neural monitoring (IONM), this study investigates its key focuses, challenges and solutions, verifies the reproducibility of the established porcine model in rats, and evaluates the feasibility of the SD rat model as an alternative to the porcine model.

**Methods:**

Twenty-four SD rats weighing 200–250 g were selected. Anesthesia was induced with 3%–5% isoflurane in oxygen at a flow rate of 1 L/min, and then maintained with 1.5%–2.5% isoflurane in oxygen at a flow rate of 0.2–0.4 L/min. Following cervical depilation, surgical disinfection, and midline cervical incision, surgeons exposed the trachea, thyroid gland, and RLN. Rats were randomized into three groups (n=8 each, 8 RLNs per group): traction injury, clamping injury, and transection injury, simulating intraoperative RLN trauma. A rat-specific electronic fiberoptic transoral laryngoscope was employed to observe vocal cord movement before and after surgery. Laryngoscopic re-examinations were performed weekly within 4 weeks after surgery, and the results were compared with the preoperative data.

**Results:**

Preoperatively, all rats had normal bilateral vocal cord movement. Within 3 postoperative weeks, all groups showed unilateral vocal cord paralysis or incomplete glottic closure. At 4 weeks, vocal cord function fully recovered in the traction group and a small number of the clamping group, but remained impaired in most clamping rats and all transection rats. All injuries caused reduced or lost RLN electromyography (EMG) signals, electrophysiological recovery was consistent with vocal cord improvement.

**Conclusions:**

Preliminary data showed similar EMG trends after traction injury in SD rats and porcine models, supporting its potential as an alternative model for further validation. The SD rat model has advantages of fast modeling and low cost, and its challenges including surgical difficulty, IONM and laryngoscopic issues can be solved by optimized surgical methods.

## 1 Introduction

This study was reported in accordance with the TITAN guidelines (1). RLN injury may occur during thyroid surgery, leading to vocal cord paralysis (2), which causes postoperative symptoms such as cough, hoarseness, dyspnea, and glottic obstruction. These symptoms severely affect the quality of life and even endanger the patient’s life. Currently, the most common mechanism of intraoperative RLN injury is not accidental transection or clamping, but traction injury to the RLN surrounded by dense fibrous tissue during thyroid lobe retraction. Regardless, avoiding acute intraoperative RLN injury remains critical.

Selective exposure and routine visual identification of the RLN during thyroid surgery are regarded as the gold standard, which significantly reduces the incidence of nerve injury. Although visual identification ensures RLN integrity, injury still occurs due to anatomical variations that sometimes make complete visual identification difficult. Studies (3–5) have shown that using IONM during thyroidectomy is associated with a significant reduction in the risk of permanent RLN injury. However, the effectiveness of IONM in preventing acute RLN injury during surgery remains controversial (6). Consequently, RLN injury animal models are being refined for better simulation of surgical conditions and improved validity.

Porcine models of RLN injury are widely used in IONM research due to the anatomical similarity of the larynx between pigs and humans, ease of tracheal intubation during surgery, and convenience of postoperative laryngoscopic imaging. Previously, Wu et al. (7) established a model using Duroc-Landrace piglets to demonstrate the optimal intensity and safety of neural electrical stimulation during IONM. Wu, Lee, Brauckhoff et al. (8–10) successively used continuous intraoperative nerve monitoring (C-IONM) to study EMG changes in a porcine model of persistent RLN traction injury. Lin, Kwak et al. (11, 12) combined a continuous IONM system to establish a porcine model of RLN thermal injury, evaluating the safe use of energy devices near the RLN during thyroidectomy. Additionally, Zhao, Zhao, Wu et al. (13–15) have continuously utilized established porcine models to conduct innovative research on laryngeal EMG recording electrodes in IONM. However, pigs as RLN injury models have several drawbacks: high procurement costs, challenges in obtaining and complying with animal ethics protocols, significant individual variations in key parameters such as body weight, gender, and health status, and demanding postoperative feeding conditions that complicate follow-up.

Therefore, there is growing interest in identifying an animal model that overcomes the limitations of porcine models. According to previous studies, rat models based on IONM (16, 17) are expected to offer greater convenience. Rat models can effectively circumvent the drawbacks of porcine models, such as difficult follow-up and high costs. Furthermore, compared with large animals, SD rats are easier to acquire, house, and observe. They also have long-recognized standardized sources and relatively minimal individual variation—factors that, if the model is successfully established, will be more conducive to electrophysiological research on the RLN. However, no established rat model of RLN injury based on IONM existed prior to this study, resulting in a lack of comparable data. In addition, performing surgical procedures on rat models presents significant technical challenges, and obtaining laryngoscopes suitable for rats has proven relatively difficult. This study therefore sought to address the following objectives. Identify the key challenges and focus areas in establishing a rat RLN injury model, and explore corresponding solutions. Utilize the established rat model combined with IONM technology to reproduce the experimental findings previously validated in porcine models—such as investigating changes in EMG signals during acute RLN traction injury and obtaining relevant baseline parameter data, the study aims to establish baseline electrophysiological parameters (e.g., amplitude, latency) for the SD rat RLN under IONM, thereby providing a foundational reference for future research. These experiments will also contribute to the development of an innovative animal model of RLN injury based on IONM.

## 2 Methods

**Figure 1:**
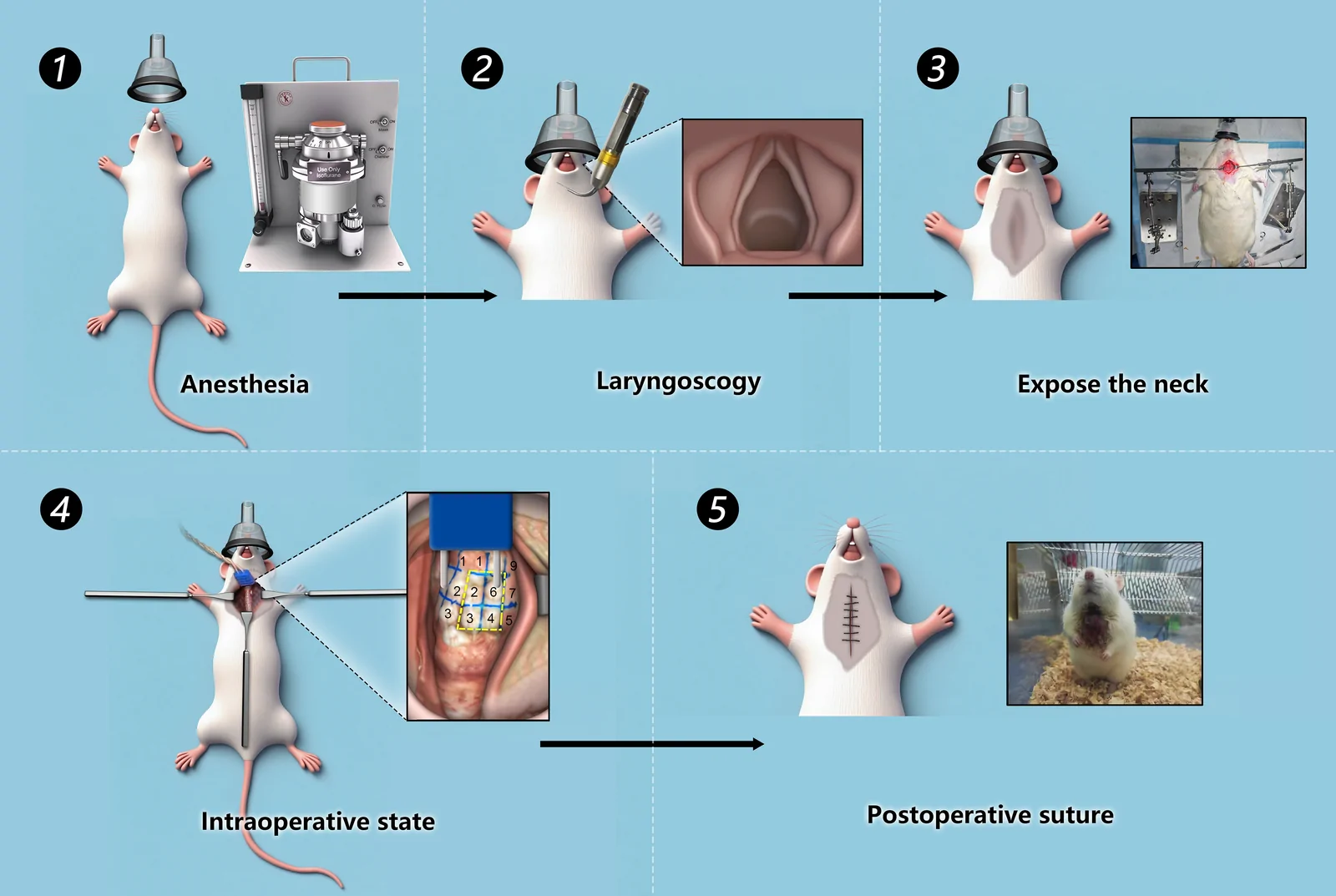
Flow chart of the experiment.

**Figure 2:**
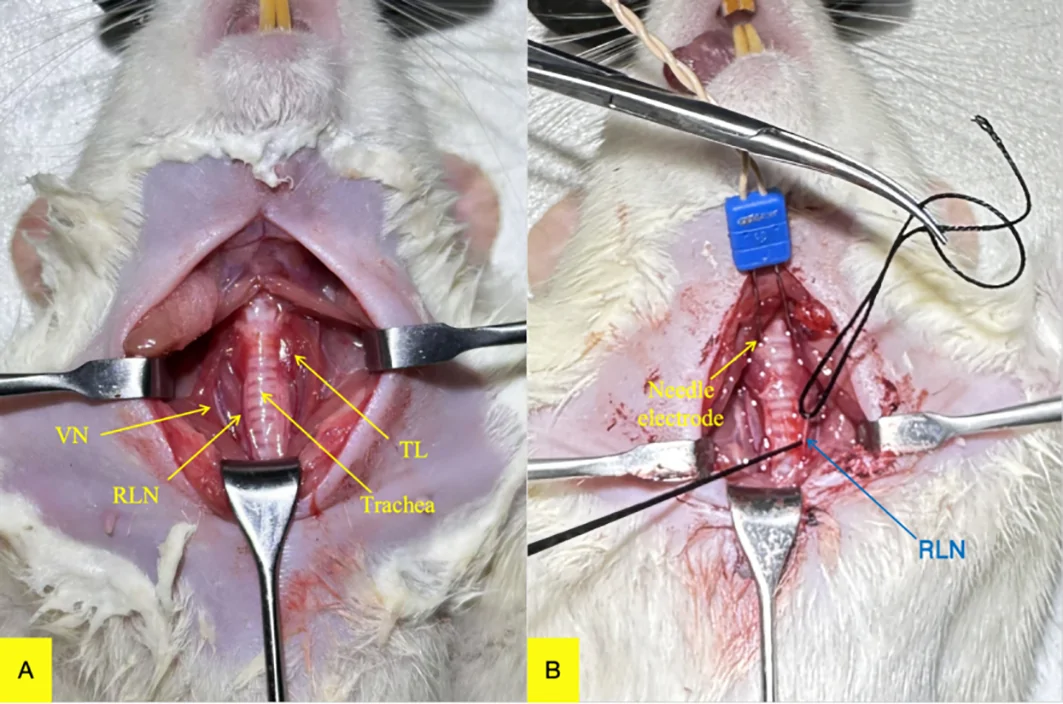
**(A)** Anatomical diagram of the rat neck. **(B)** Simulation of recurrent laryngeal nerve traction injury.

The work has been reported in accordance with the ARRIVE guidelines (Animals in Research: Reporting *In Vivo* Experiments) (18). The study protocol was approved by the Institutional Animal Care and Use Committee of the Laboratory Animal Center of Jilin University (China). (Ethics number: KT20250293). **Figure 1** depicts the workflow of the entire experiment. For rat experiments, the sample size should be no fewer than 6 animals to meet basic statistical requirements. 24 SD rats weighing 200–250 g. 24 rats were numbered 1–24 in order of body weight and equally divided into three groups using a completely random method. The specific procedure was as follows: 24 small slips of paper were prepared—8 labeled “Group A,” 8 labeled “Group B,” and 8 labeled “Group C.” These slips were placed into an opaque container and thoroughly mixed. The rats were then assigned by drawing slips sequentially according to their numbered order (1–24). The entire grouping process was performed by personnel not involved in the subsequent experiments. Surgical procedures were carried out by the second author, who was aware of only the operational steps and not the group assignments or their experimental significance. Outcome assessments were performed by research assistants who were blinded to the group allocations. Data analysis was conducted by the first author using blinded data (coded as Group 1, Group 2, etc.). The corresponding author performed unblinding only after all statistical analyses were completed. This study was designed as a pilot proof-of-concept investigation to evaluate the feasibility of establishing an RLN injury model in SD rats using IONM. Therefore, a small sample size (n=6) was selected to focus on model development, technical challenges, and initial signal characterization rather than on statistical hypothesis testing at first. Subsequently, considering that the small sample size was a major limitation of this study. We repeated the experiment using 24 rats. A total of 24 animals were included in the final analysis. This is a combined dataset with a final sample size of n=24, rather than two separate or independent cohorts. After inducing anesthesia with 3%–5% isoflurane in oxygen (flow rate: 1 L/min), the SD rats were placed in a supine position on the operating table with their necks extended. A rat-specific electronic fiberoptic transoral laryngoscope (parameters: optical fiber diameter = 3 mm, tongue depressor length = 25 mm, tongue depressor width = 5.5 mm) was used. The laryngoscope was advanced slowly along the right side of the tongue base to the root of the tongue, and the epiglottis was gently lifted to expose the glottis. Changes in the vocal cords during rat respiration were observed to confirm normal bilateral vocal cord movement. Anesthesia was then maintained with 1.5%–2.5% isoflurane in oxygen (flow rate: 0.2–0.4 L/min) to ensure a stable general anesthetic state. After removing the hair from the rat’s neck using depilatory cream, the rat was kept in a supine position with the neck extended. The surgical area was disinfected three times with 2% povidone-iodine, followed by iodine neutralization with 75% ethanol. The surgeon made a 2 cm skin incision along the midline of the anterior neck. After separating the subcutaneous tissue, further dissection was performed to expose the muscle layer. The cervical muscles were separated longitudinally to reveal the trachea and thyroid gland. The second assistant used small eyelid retractors to retract the neck tissues bilaterally, while the first assistant used medium-sized eyelid retractors to retract downward, ensuring adequate exposure of the neck and laryngeal regions. Needle-type recording electrodes were carefully inserted into the middle-lower region of the anterolateral angle of the thyroid cartilage, with the insertion depth controlled to less than 2 mm to prevent potential damage to the underlying laryngeal tissues. The surgeon clamped the distal end of the thyroid gland with ophthalmic forceps and gradually separated the thyroid tissue from the trachea. The anterior attachments of the thyroid gland were carefully dissected; after clamping and hemostasis, exposure or removal of the thyroid gland was completed (**Figure 2A**). The NIM 3.0 neural monitoring system was used to record real-time EMG signals. The first assistant used a handheld monopolar stimulation probe to identify the bilateral vagus nerves and RLNs. When a threshold of 100 microvolts (μV) was reached, the event capture function was activated. The surgeon carefully dissected and separated the tissues surrounding the nerves. The latency and initial amplitude after probe stimulation were recorded and used as baseline control data. Signal loss (LOS) was defined as either an interruption of the EMG signal or an EMG signal amplitude below 100 μV despite an initially intact signal. The threshold for adverse events related to RLN injury was set at approximately 50% of the initial EMG signal amplitude.

This study included 24 SD rats (involving 24 RLNs), which were randomly divided into three groups based on injury mechanism: Traction Injury Group (8 rats, 8 RLNs), Clamping Injury Group (8 rats, 8 RLNs), and Transection Group (8 rats, 8 RLNs). The study aimed to observe changes in EMG signals induced by acute RLN injury via different mechanisms in each group.Traction Injury Group;A 1–0 surgical suture was looped around the RLN and secured with a curved hemostat (**Figure 2B**). A dynamometer was used to apply 0.61 N of traction force to the nerve. Traction was stopped under one of the following conditions: signal loss (LOS), signal impairment, or when the traction duration exceeded 1 minute. EMG signals and latency were recorded before and immediately after traction. EMG signals and latency were re-evaluated 4 weeks post-injury. This group simulated RLN injury caused by traction on the thyroid lobe during thyroid resection (a common scenario in clinical surgery). Clamping Injury Group:An hemostat was used to clamp the RLN. EMG signals and latency were recorded immediately after clamping. EMG signals and latency were re-monitored 4 weeks post-injury. This group simulated accidental RLN clamping during surgery, which typically occurs due to inadequate exposure or visual misidentification of the nerve. Transection Group:One side of the RLN was transected. EMG signals and latency were recorded immediately after transection. EMG signals and latency were re-assessed 4 weeks post-injury. This group simulated accidental RLN transection during surgery, also resulting from poor exposure or incorrect visual identification.

After completing the experimental procedures, the rats underwent layered suturing of the muscular layer, subcutaneous tissue, and epidermis. The muscular layer and overlying fascia were sutured with 4–0 chromic gut suture. The skin was closed with 5–0 nylon suture. The anesthetic gas was switched to pure oxygen to accelerate the metabolism of residual anesthetics. Mask oxygen supply was maintained for 1–2 minutes, and the rats were monitored until their respiratory rate returned to normal.

During the 4-week postoperative observation period (no interventions were applied), laryngeal images of vocal cord movement were captured weekly. These images were compared with preoperative images to evaluate vocal cord function—only whether vocal cord movement was “intact” or “impaired” was recorded. Conventional laryngoscopy allows for direct observation of the anatomical structures of the throat and the morphology of lesions, while the glottal closure pattern is one of the key aspects of this evaluation, among which irregular closure is defined as the presence of an irregular linear gap in the glottis (19).

In this experiment, the compression pressure on the nerve was indirectly standardized by controlling three physical variables: a constant traction force, a uniform suture specification, and a consistent traction direction (perpendicular to the long axis of the nerve). By holding these three parameters constant across all animals, the local pressure applied to the nerve was effectively identical among all subjects. The present study was designed to investigate the injury effects at this specific force level, rather than to explore a dose-response relationship. Therefore, a gradient design was not incorporated in the experimental protocol. During the procedure, the depth of anesthesia was indirectly assessed by continuous monitoring of mean arterial pressure (MAP) and heart rate.

During the preparation of this manuscript, the authors used Doubao (Doubao, developed by ByteDance, based on the Doubao large language model, web version) for language polishing of the draft. Specifically, the AI tool was applied to refine sentence fluency, improve word choice. All AI-generated content was critically reviewed, substantially revised, and edited by the authors. The authors take full responsibility for the final content, interpretation of data, and conclusions presented in this manuscript. No AI tool was used for data collection, statistical analysis, experimental design, or the generation of any figures or tables. The proportion of AI-assisted content in the initial draft is estimated to be approximately 10%, and more than 90% of the final manuscript text represents original author composition and manual revision.

## 3 Results

Comparison of Vocal Cord Movement Before and After Surgery. Preoperatively, laryngeal movement images of all rats were acquired using a rat-specific electronic fiberoptic transoral laryngoscope. The epiglottis exhibited a normal morphology and structure with a smooth mucosa; meanwhile, the bilateral vocal cords showed complete closure without any abnormal movement. During the postoperative observation period, laryngoscopic examinations were performed weekly. Within 3 weeks postoperatively, unilateral vocal cord paralysis or incomplete glottic closure was observed in all surgical groups. Four weeks postoperatively, vocal cord paralysis or incomplete glottic closure had recovered completely in a small subset of the clamping injury group and all rats in the traction injury group, whereas no recovery was observed in the majority of the clamping injury group or in the transection group.

Changes in EMG Signals During Continuous Nerve Monitoring of RLN Traction Injury. A total of 24 RLNs were involved in this surgical procedure, and induced changes in EMG signals were observed in all experiments. Changes in EMG signal amplitude and latency of the 24 RLNs are presented in **Table 1**. Traction injury resulted in variable EMG amplitude reduction and signal loss in some nerves, with amplitude recovery observed at 4 weeks in surviving nerves. Transection led to sustained signal loss. For clamping injury, amplitude reduction was observed with recovery at 4 weeks in some nerves, while others resulted in persistent loss of EMG signals.

**Table 1:** Signal data by different injuries.

Damage model	Subject (side)	Force, N	Time of duration, s	Baseline EMG		EMG after injury			EMG after postoperative 4 weeks			
				Amplitude, μV	Latency, ms	Amplitude, μV	Latency, ms	Loss of signal	Amplitude		Latency	
									μV	mean(%)	ms	mean(%)
Traction injury												
	1(L)	0.61	60.0	1209.0	1.000	1081.0	1.000	No	1190.0	98.4	1.000	100
	2(L)	0.61	60.0	1450.0	1.000	1311.0	1.000	No	1445.0	99.7	1.000	100
	3(L)	0.61	60.0	2551.0	1.000	1204.0	1.000	Latency only	1767.0	69.3	1.000	100
	4(L)	0.61	60.0	204.0	1.000	150.0	1.000	No	167.0	81.9	1.000	100
	5(L)	0.61	53.0	1856.0	1.000	—	—	Loss of signal	1115.0	60.0	1.000	100
	6(L)	0.61	60.0	2135.0	1.000	1181.0	1.000	Latency only	1926.0	90.2	1.000	100
	7(L)	0.61	60.0	1681.0	1.000	1483.0	1.000	No	1483.0	88.2	1.000	100
	8(L)	0.61	56.0	746.0	1.000	—	—	Loss of signal	469.0	62.9	1.000	100
Clamp injury												
	9(L)	half*	5.0	1152.0	1.000	—	—	Loss of signal	1098.0	95.3	1.000	100
	10(L)	complete*	5.0	1069.0	1.000	—	—	Loss of signal	—	—	—	—
	11(L)	half	10.0	2428.0	1.000	—	—	Loss of signal	2186.0	90.0	1.000	100
	12(L)	complete	10.0	2439.0	1.000	—	—	Loss of signal	—	—	—	—
	13(L)	half	30.0	1115.0	1.000	—	—	Loss of signal	—	—	—	—
	14(L)	complete	30.0	1483.0	1.000	—	—	Loss of signal	—	—	—	—
	15(L)	half	60.0	3598.0	1.000	—	—	Loss of signal	—	—	—	—
	16(L)	complete	60.0	1900.0	1.000	—	—	Loss of signal	—	—	—	—
Transection												
	17(L)	—	—	1317.0	1.000	—	—	Loss of signal	—	—	—	—
	18(L)	—	—	1204.0	1.000	—	—	Loss of signal	—	—	—	—
	19(L)	—	—	1767.0	1.000	—	—	Loss of signal	—	—	—	—
	20(L)	—	—	1096.0	1.000	—	—	Loss of signal	—	—	—	—
	21(L)	—	—	1859.0	1.000	—	—	Loss of signal	—	—	—	—
	22(L)	—	—	2051.0	1.000	—	—	Loss of signal	—	—	—	—
	23(L)	—	—	2139.0	1.000	—	—	Loss of signal	—	—	—	—
	24(L)	—	—	1680.0	1.000	—	—	Loss of signal	—	—	—	—

EMG, electromyography; L, left; R, right.

*half: Clamp a portion of the nerve's cross-section with a vascular clamp; complete: Clamp the cross-section of the nerve with a vascular clamp.

“—” indicates loss of signal. In this experiment, the primary observation data is signal amplitude. Therefore, when the amplitude drops below 100 μV, the corresponding data are marked with “—”.

**Figure 3:**
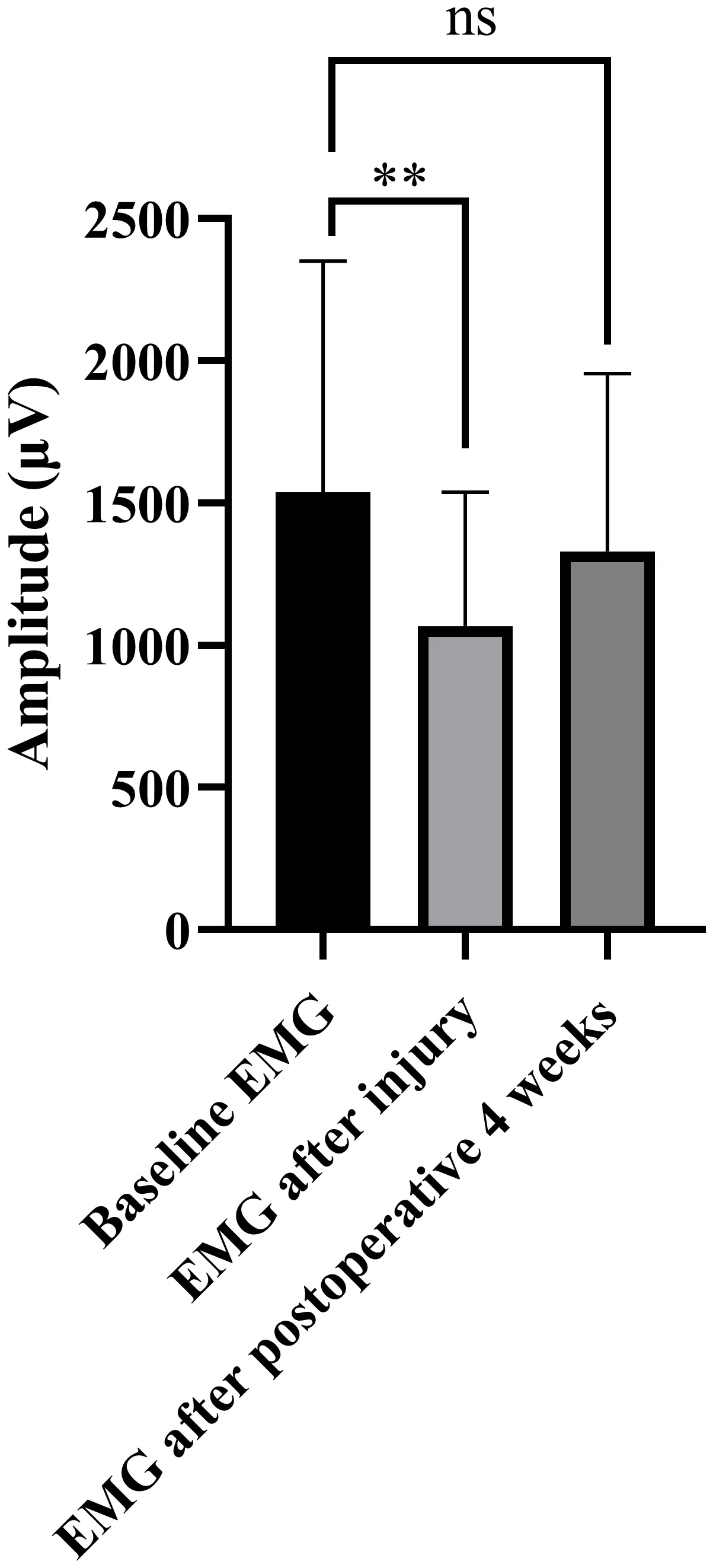
The amplitude differences between the post-injury and four weeks post-injury and the pre-injury.

Statistical Analysis: In the traction injury model, six nerves with complete paired data were analyzed. Given the small sample size and the fact that the normality assumption for the differences was not met (Shapiro-Wilk test, P = 0.04), the Wilcoxon signed-rank test was used for within-group comparison. The results showed that the EMG amplitude at 4 weeks postoperatively (mean ± standard deviation: 1329.7 ± 625.3 μV; median: 1464.0 μV) was significantly higher than that immediately after injury (1068.3 ± 470.2 μV; median: 1192.5 μV), with the difference being statistically significant (Z = -2.023, P = 0.043). All analyses were performed using SPSS software. Data from the clamping and transection groups are presented as preliminary qualitative observations. **Figure 3** shows a bar chart generated using GraphPad Prism software for difference analysis.

## 4 Discussion

It is assumed that the experimental methodology established in porcine models can be effectively transferred to other animal models. This assumption has been verified in the SD rat model, which exhibits good adaptability in this study. Experiments have confirmed that in the SD rat model, when intraoperative neuromonitoring is applied to simulate nerve injury scenarios, the variation trend of EMG signals is consistent with that of the pig model. However, compared with the pig model (9),the overall amplitude of EMG signals in the SD rat model is relatively higher. Preliminary data indicate that the electromyographic changes observed after traction injury in the pig model show a similar directional trend, suggesting that SD rats have potential as alternative subjects for further validation.

### 4.1 Discussion of results

The RLN waveform in SD rats is a sharp, high-amplitude biphasic wave with short latency, reflecting rapid depolarization/repolarization and matching their high-pitched vocalizations and short nerve length. In humans, the biphasic waveform is more rounded and gentle, with lower amplitude, corresponding to finer vocal control and lower vocal cord vibration frequency. The pig’s waveform is intermediate, with moderate amplitude and shorter latency than humans, aligning with its vocalization frequency and respiratory demands, as well as its shorter neck (**Table 2**).

**Table 2:** Waveforms comparison.

Comparison object	SD rats	Experimental pigs	Human
waveforms	biphasic waves	biphasic waves	biphasic waves
amplitude	high	fall in between	low
latent period	short	fall in between	long
waveform corners	sharp waves	fall in between	rounded waves

The average amplitude after injury was lower than that before injury, which is consistent with the nerve state post-injury. Four weeks after injury, the average amplitude recovered, presumably related to the injury and subsequent repair of the nerve. Regarding the higher EMG signal amplitude in SD rats, it may be associated with muscle fiber type (20): SD rats are dominated by fast-twitch muscle fibers, which exhibit high action potential firing frequency and large amplitude. In contrast, pigs have a higher proportion of slow-twitch muscle fibers, resulting in relatively gentle EMG signals. This amplitude difference may also be linked to body size factors: the trapezius muscle in the neck of SD rats is small, so the electrode implantation area is concentrated, making it easy to capture high-amplitude signals. Pigs, however, have larger muscle groups and longer electrode spacing, which requires adjustment to avoid signal attenuation. The specific impact of the amplitude difference is currently unknown, and there is no relevant research available; further investigation is therefore required.

Regarding the possible causes of the extreme variability observed in the baseline EMG data. First, the nerve may have been slightly compressed by adjacent blood vessels or tissues, affecting signal conduction. Second, measurement system errors may have occurred, as the nerve signals could have been influenced by factors such as the depth of anesthesia and physiological changes over time during the measurement.

### 4.2 Advantages of SD rat model for RLN injury

SD rats exhibit a high reproductive rate, low breeding cost, and modest survival requirements. In contrast, pigs have a long reproductive cycle, with a gestation period of approximately 115 days (21), and reach sexual maturity at 6–8 months of age. SD rats, however, have a gestation period of only 21–23 days and achieve sexual maturity at around 6–12 weeks (22). The rapid reproductive capacity of SD rats enables the quick provision of a large number of experimental samples, meeting the needs of rapid experimentation and repeated trials. With the same experimental cost, using SD rats allows for more sufficient and convincing experimental data to be obtained. Compared with the favorable postoperative survival rate of SD rats, the pig model has a significantly higher perioperative mortality rate, which seriously affects the postoperative observation period and the feasibility of repeated surgical procedures. For example, after conducting *in vivo* experiments on pig models, Lee (9) and colleagues could only perform subsequent experiments *in vitro*. In other research fields (23), postoperative follow-up of pig models is also limited to simple assessments, which further confirms the high difficulty of follow-up for pig models.

SD rats are subject to fewer ethical and regulatory restrictions. Pigs, with their physiological structure and metabolic system highly similar to those of humans, hold unique value in research on the cardiovascular system, digestive system, and other fields. However, precisely because of this similarity, using pigs for experiments faces greater ethical controversies and strict regulatory requirements. In contrast, as small rodents, SD rats are subject to relatively lenient ethical review for experimental use. Their usage standards comply with the animal experiment guidelines of most countries. Researchers do not need to spend excessive energy dealing with complex ethical approval processes when conducting experiments, allowing them to devote more time and resources to the actual research. the SD rat model has been widely applied in the field of thyroid research (24–26).

SD rats are classified as an outbred stock with a well-documented breeding history and relatively low phenotypic variability among individuals maintained under standardized conditions (27). As an outbred stock, SD rats retain substantial genetic heterogeneity rather than the genetic uniformity characteristic of inbred strains. Nevertheless, long-term controlled breeding under defined management protocols has produced populations with stable phenotypic traits (28). Research data shows that the dispersion of body weight and physiological indicators among SD rats from the same batch is less than 5%. This phenotypic stability helps to minimize experimental variability and supports the reproducibility of research findings. In practical terms, if an SD rat dies accidentally during an experiment, another animal of the same strain, age, sex, and comparable body weight can often be substituted as a replacement. In contrast, if an experimental pig dies during the study, finding a replacement individual with sufficiently matched physiological status and body parameters is considerably more difficult. As outbred large animals, pigs exhibit greater inter-individual genetic and phenotypic variation, making it challenging to obtain a well-matched substitute within a short timeframe. Replacing a pig in an ongoing experiment may introduce confounding variables, potentially compromising data reliability and experimental continuity.

SD rats are docile in temperament (27) and exhibit strong adaptability to the environment. After surgical procedures, pigs, due to their large body size and intense stress response, pose high challenges in nursing care, which may compromise the accuracy and completeness of experimental data. In contrast, SD rats are easy to handle and manipulate. This enables the acquisition of comprehensive, systematic, and reliable experimental data.

### 4.3 Other optimization advantages of SD rat model for RLN injury

In the establishment of animal experimental models, there are significant differences in preoperative preparation between pigs and SD rats. For pig experimental models, combined anesthesia is usually required (29, 30), and the process is relatively complex. In contrast, SD rats only need inhalational anesthesia (31), which is less difficult to maintain during surgery, and no muscle relaxants are needed. Regarding the issue of fixing SD rats during preoperative preparation, we can optimize the binding method: use soft and elastic restraint straps to fix the rats stably on the operating table. Considering the small size of SD rats, the operating table needs to be modified specifically. The size of the tabletop should be reduced to ensure fixation effectiveness and operational safety; stainless steel or corrosion-resistant plastic should be used as the material to facilitate high-frequency disinfection (since SD rats have strong disease resistance, but cross-infection prevention is still necessary).

### 4.4 Limitations and solutions of the SD rat model for RLN injury

Despite the aforementioned advantages of the SD rat model, it also has limitations compared with the pig model. Below, these limitations are enumerated, and corresponding solutions are proposed.

#### 4.4.1 Surgical difficulty

Adult SD rats have a body length of 18–20 cm, and combined with their narrow, elongated heads, this significantly increases the difficulty of surgical operations, resulting in a limited operable space. The target anatomical structure—the RLN—has an average transverse diameter of only approximately 1.95 mm and appears silvery white. It runs in the groove between the trachea and esophagus. Due to its small size, the nerve is easily confused with surrounding fascia or blood vessels under direct naked-eye observation. Considering the anatomical structure, the right RLN loops around the right subclavian artery and is adjacent to structures such as the superior vena cava and azygos vein arch. The narrow space here makes it easy to damage blood vessels and cause bleeding during operation. The left RLN ascends after looping around the aortic arch; it is located relatively deep and mostly runs behind the inferior thyroid artery, making it prone to accidental ligation of the artery or traction injury to the nerve during surgery.

In response to the above issues, we need to focus on the following key operational points. During the surgical field exposure phase, depilatory cream was first used to thoroughly remove the hair on the neck of SD rats. Then, the rats were fixed in a supine position and their necks were fully extended. After strictly disinfecting the surgical area three times with strong iodine, a 2cm longitudinal incision was made along the anterior midline of the neck. This length can precisely cover the area from the thyroid cartilage to the sternal stalk. It can not only ensure that the surgical field is fully exposed, but also reduce unnecessary tissue damage, creating favorable conditions for the anatomy of the RLN. Because the thyroid gland of SD rats is very small in size, during the exposure of the RLN, there is no need for thyroidectomy. Just gently pulling it to one side can effectively avoid iatrogenic damage to the RLN caused by thyroidectomy. Given the differences in the anatomical structures of the left and right sides of rats, the separation strategy needs to be adjusted specifically: On the right side, due to the more complex distribution of blood vessels, the blood vessels should be dealt with first; On the left side, a stratified passive separation technique is adopted to reduce the risk of damage to the neural tissue. In addition, it is recommended to adopt microsurgical techniques. With the assistance of microscopic instruments and magnification equipment, the accuracy of RLN recognition can be enhanced. Combined with electrophysiological detection technology, precise nerve positioning can be achieved, further ensuring the safety and effectiveness of surgical operations. (Refer to **Figure 2A**).

#### 4.4.2 Intraoperative nerve monitoring

IONM stimulates the RLN via a probe, collects the signals (EMG) generated when the motor nerve fibers of the RLN conduct to the muscles, and forms waveforms and prompt sounds on the RLN monitor. The analysis of such electrophysiological signals can reflect the functional status of the nerve in real time.

The intraoperative RLN monitoring technology in humans realizes real-time monitoring of nerve function through a special endotracheal tube integrated with electrodes. Its core principle lies in the construction of an electrophysiological signal closed-loop system: the electrodes embedded in the endotracheal tube that contact the vocal cords capture electromyographic signals, forming a dual mechanism of active detection and passive monitoring. This technology improves the accuracy of anatomical localization to the millimeter level. Through dynamic signal analysis and postoperative functional verification, it greatly enhances the accuracy of nerve identification and reduces the rate of nerve injury. In clinical application, interference from muscle relaxants must be strictly avoided.

In both pig and SD rat models of RLN injury, endotracheal intubation can be applied to simulate that used in human thyroid surgery, thereby enabling nerve monitoring. Although there are differences in the specific design of endotracheal tubes among the three (humans, pigs, and SD rats), their functional principles are identical. Focusing on the SD rat model, the small body size of SD rats increases the difficulty of endotracheal intubation, which in turn raises the technical challenge of IONM.

To effectively address the current challenges, a literature review was conducted, and the following three solutions were summarized. Solution 1 focuses on improving the intubation technique, specifically modified blind endotracheal intubation. The rat is placed in a supine position, and the middle finger is used to support the highest point of the rat’s parietal bone. The thumb is then used to palpate the laryngeal region of the rat’s airway, aligning the pharynx and trachea at the same horizontal level. Zheng et al. (32) have demonstrated that this method is safer, faster, and simpler. Solution 2 emphasizes the use of auxiliary equipment. Jin et al. (33) developed a supraglottic intubation assist catheter (SIAC), which has been further optimized; the success rate of intubation using this device has reached 100%. Solution 3 focuses on innovating monitoring technology, utilizing transcartilaginous surface recording electrodes for IONM. During surgery, the protection of nerve function is crucial, as even a slight mistake may lead to irreversible postoperative nerve injury in patients. With its unique working mechanism, transcartilaginous surface recording electrodes can capture changes in nerve electrical signals in real time and with high precision during surgical manipulation. The electrode needle is inserted on the surface of the cartilage, and based on electrophysiological principles, nerve activity is converted into observable and analyzable electrical signal data. Using this data, surgeons can clearly understand the functional status of nerves in the surgical area, promptly identify potential risks of nerve injury, and quickly adjust surgical strategies. This minimizes interference and damage to nerves to the greatest extent, achieving both improved surgical safety and therapeutic efficacy. While handheld probe electrodes are operator-dependent and surface electrodes can be affected by muscle relaxants, needle electrodes provide superior signal stability and interference resistance.

When adopting transcartilaginous surface recording electrodes in Solution 3, attention should be paid to the insertion position and depth of the electrode needle, as well as the risk to the SD rat’s life. Solution 3 was selected for this study (refer to **Figure 2B**). By referring to the research by Zhao et al. (14) on the effect of needle recording electrode position on nerve signal amplitude, we can roughly determine the insertion position of the experimental electrode. Meanwhile, Zhao et al.’s study indicates that the insertion depth of the electrode needle has no significant effect on signal amplitude. Based on this finding, we can adhere to the “minimal invasion” principle under the premise of ensuring stable signal acquisition, minimizing the insertion depth as much as possible. This operation not only effectively avoids the risk of damaging important organs and tissue structures of the rat but also reduces the incidence of postoperative complications such as infection and tissue adhesion, laying a foundation of a healthy animal model for subsequent long-term nerve signal monitoring and behavioral experiments. Additionally, the rat’s vital signs should be monitored in real time during the experiment. A multi-parameter monitor should be used to dynamically record heart rate, respiratory rate, and blood oxygen saturation, ensuring that both experimental safety and animal welfare requirements are met.

#### 4.4.3 Laryngoscopic examination and laryngoscopic follow-up before and after surgery

In animal model research, preoperative and postoperative laryngoscopic examinations have become a core method for evaluating vocal cord function, thanks to their intuitive visualization advantages. By dynamically observing the coordination of vocal cord opening and closing, it is possible to accurately determine the presence of abnormalities such as vocal cord paralysis and limited movement. Meanwhile, comparative analysis of postoperative laryngoscopic images with preoperative baseline data can effectively identify surgery-related nerve injuries and conduct quantitative assessments of the repair process.

In studies on pig models of RLN injury, conventional laryngoscopes are used for preoperative examination and postoperative follow-up. Owing to the pig’s suitable body size, these procedures are easy to perform and provide a clear field of view. However, in studies involving small experimental animals like SD rats, traditional laryngoscopes are difficult to meet the examination requirements, restricted by the rats’ tiny laryngeal structures and limited operational space. Thus, innovative solutions are urgently needed.

Samsamshariat et al. (34) successfully performed rat endotracheal intubation safely with a high success rate using a 0.035-inch straight guidewire for guidance and a size 0 Miller-type infant laryngoscope blade, without the need for atropine. In addition to this method, we propose the development of a miniaturized laryngoscope specifically designed for SD rats. This device will be closely tailored to the anatomical characteristics of the rat larynx, with customized design of the laryngoscope body’s length, diameter, and bending angle to ensure precise exploration within the narrow space. In terms of functional design, emphasis will be placed on enhancing real-time high-definition dynamic imaging performance to facilitate continuous observation of vocal cord movement. At the same time, non-essential functions such as biopsy and foreign body removal will be appropriately simplified. On the premise of ensuring core detection efficiency, the device’s size and operational convenience will be optimized, providing efficient and reliable technical support for the evaluation of laryngeal function in SD rats before and after surgery.

#### 4.4.4 The limitations of this study beyond the surgical procedure

We acknowledge several limitations in the present study. First, at the technical level, the small size of the rats and the fragility of the related neural structures necessitated the execution of all procedural steps with extreme precision and care, imposing high demands on operational accuracy. Second, in terms of sample size, as this study represents an exploratory pilot experiment, the initial planning of experimental animal numbers was not fully adequate. Last but not least, as the lifespan of a rat is comparatively short, studying the long term effects of RLN injuries would not be possible using rats. However, in this study, damage to some nerves is recoverable, and any nerve damage that persists beyond the rat’s lifespan is generally irreversible.

In response to the above issues, we propose the following improvement strategies. First, at the technical level, we intend to introduce magnification equipment, such as magnifying lenses or endoscopic systems, to significantly enhance intraoperative visualization, thereby enabling precise identification and protection of neural structures and minimizing non-interventional damage. Second, in terms of experimental design, we have already implemented improvements by conducting an in-depth study with an expanded sample size, using 24 rats (24 nerves in total), to address the limitations of the current pilot study in statistical power and the generalizability of conclusions.

We emphasize that this pilot study establishes the feasibility of creating an IONM-guided RLN injury model in SD rats. Traction injury showed a pattern of EMG recovery similar to larger models, while preliminary observations suggest sustained deficit after clamping or transection. The model presents technical challenges but offers cost and logistical benefits. Future studies with larger, balanced cohorts are required to robustly characterize injury-specific electrophysiological and functional outcomes and validate the model against porcine standards.

### 4.5 Postoperative EMG evaluation method for rats

Tessema B et al. (17) developed a standardized minimally invasive transoral laryngeal bipolar electromyography (ToL EMG) method to assess RLN recovery after controlled crush injury in rat models (35). The animals were placed in a supine position on a modified stereotaxic operating table tilted at 15°. A 3–0 silk suture was used to suture the tongue at the midline of its anterior third and suspend it, retracting the tongue. A laryngoscope was inserted transorally to expose the laryngeal interior and provide clear visualization of the glottic region. Vocal cord movement was observed by the researchers. Postoperatively, transoral laryngeal EMG was performed to record the activity of the adductor muscles and posterior cricoarytenoid muscles on both the injured side and the control side. EMG recordings of the posterior cricoarytenoid muscles were obtained during the phasic respiratory cycle. Recordings of the adductor muscles were acquired during spontaneous adduction. All electromyographic activities were spontaneous and not evoked (36).

### 4.6 Areas for improvement of the SD rat model

Based on the above research findings, although the SD rat model has proven its feasibility and significant advantages, there are still obvious limitations to its widespread application in the field of scientific research. The main reason can be attributed to the inherent technical flaws of the model, i.e., the disadvantages mentioned in the above article. Despite the corresponding improvement schemes proposed in this study, many technical challenges remain in the practical implementation phase. Further optimization of these solutions requires continued academic discussion.

## Data Availability

The original contributions presented in the study are included in the article/supplementary material. Further inquiries can be directed to the corresponding authors.
