# Correction: Establishment of recurrent laryngeal nerve injury animal models using intraoperative neural monitoring: SD rat models

**DOI:** 10.3389/fendo.2026.1954017

**Published:** 2026-08-11

**Authors:** Yuqing Gao, Huixin Li, Tie Wang, Yishen Zhao, Shijie Li

**Affiliations:** Department of Thyroid Surgery, China-Japan Union Hospital of Jilin University, Changchun, China

**Keywords:** animal model, intraoperative neural monitoring, Recurrent laryngeal nerve injury, Sprague-Dawley (SD) rats, thyroid

The funder “Key Scientific Research Project of Education Department of Jilin Province (No. JJKH20261625KJ); Jilin Province Health Research Talent Special Project (No. 2025SCZ70)” to “Gao Y, Li H, Wang T, Zhao Y and Li S” was erroneously included.

The original version of this article has been updated.

